# Protocol for cortical-wide field-of-view two-photon imaging with quick neonatal adeno-associated virus injection

**DOI:** 10.1016/j.xpro.2021.101007

**Published:** 2021-12-10

**Authors:** Ikumi Oomoto, Hiroyuki Uwamori, Chie Matsubara, Maya Odagawa, Midori Kobayashi, Kenta Kobayashi, Keisuke Ota, Masanori Murayama

**Affiliations:** 1 Center for Brain Science, RIKEN, 2-1 Hirosawa, Wako-shi, Saitama 351-0198, Japan; 2 Section of Viral Vector Development, National Institute for Physiological Sciences, 38 Nishigonaka, Myodaiji-cho, Okazaki-shi, Aichi 444-8585, Japan; 3 Department of Neurochemistry, Graduate School of Medicine, The University of Tokyo, 7-3-1 Hongo, Bunkyo-ku, Tokyo 113-0033, Japan

**Keywords:** Microscopy, Model Organisms, Neuroscience

## Abstract

We recently established a simple and versatile adeno-associated virus (AAV) induction approach that enables dense (>90% labeled neurons) and cortical-wide Ca^2+^ sensor expression. Here, we describe the stepwise protocol for neonatal AAV injection of a Ca^2+^ sensor. We also detail the steps for subsequent craniotomy to generate a chronic cranial window, followed by wide-field two-photon Ca^2+^ imaging in an awake mouse. This protocol serves as an alternative to the use of transgenic animals and offers translatable options for cortical-wide experiments.

For complete details on the use and execution of this protocol, please refer to Ota et al. (2021).

## Highlights

• Fast, cell-resolution and wide field-of-view (FOV) Ca^2+^ imaging in an awake mouse
• Dense and cortical-wide Ca^2+^ sensor expression with quick neonatal AAV injection
• Transcranial evaluation of the Ca^2+^ sensor expression with one-photon macro imaging
• Making a chronic large cranial window for the wide FOV two-photon imaging

## Graphical abstract

**Figure:**
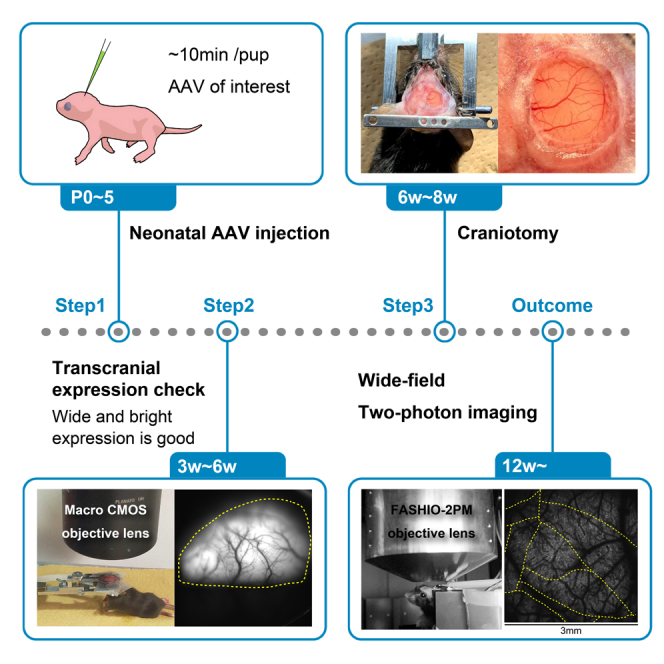

## Teaser

We recently established a simple and versatile adeno-associated virus (AAV) induction approach that enables dense (>90% labeled neurons) and cortical-wide Ca^2+^ sensor expression. Here, we describe the stepwise protocol for neonatal AAV injection of a Ca sensor. We also detail the steps for subsequent craniotomy to generate a chronic cranial window, followed by wide-field two-photon Ca^2+^ imaging in an awake mouse. This protocol serves as an alternative to the use of transgenic animals and offers translatable options for cortical-wide experiments.

## Before you begin

All animal experiments were performed in accordance with the institutional guidelines and were approved by the Animal Experiment Committee at RIKEN. C57BL/6 mice (Japan SLC, Inc., Hamamatsu, Shizuoka, Japan) were bred in our colony at RIKEN and maintained on a 12/12 h light/dark cycle (lights on 8 am to 8 pm) with *ad libitum* access to food and water. Pregnant mother mice were bred individually in each cage. The adeno-associated virus (AAV) injection protocol can be initiated once the pups are born (postnatal day 0, P0).

### Mating

**Timing: 30 min (approximately 3 weeks before the experiment)**

1. Mate the mice of your interest at least 3 weeks before the experiment.

***Note:*** In our laboratory, we set up a breeding cage by adding two or three healthy females with a single resident male.

2. When the pregnancy is confirmed, isolate the pregnant female by placing her in a clean cage with fresh nesting material and a covered shelter where she can hide.

***Note:*** By setting up a covered shelter in the home cage, the mother mouse can feel safe and take better care of the pups, which increases their survival rate.

***Optional:*** Pregnant mother mice may be purchased from the breeder.

### Preparation of glass pipettes for injection

**Timing: 1 h**

3. Prepare appropriate quartz glass pipettes using a laser puller.
  a. The laser puller settings are presented in a table in the materials and equipment section.

**Figure 1:**
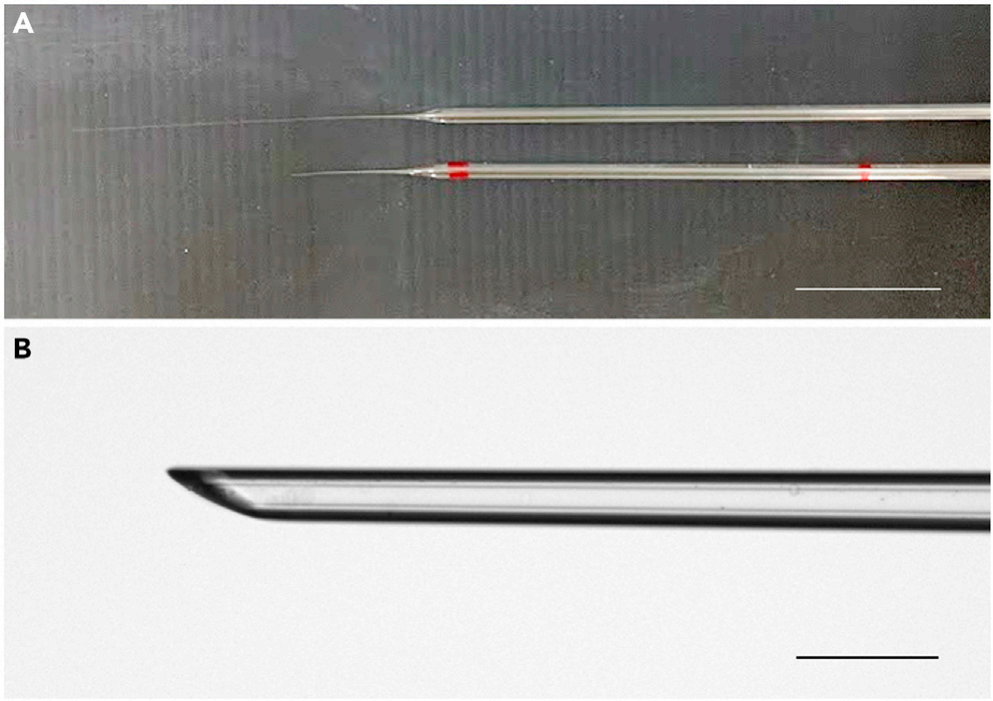
Glass pipettes used in this protocol (A) Top: The glass pipette after pulling using the laser puller. Bottom: The glass pipette after cutting the tip. The red marks on the pipette are markers for the injection volume. (The marker at 5.68 cm is not shown). (B) The magnified view of the glass pipette tip after cutting and polishing. The length of the tip should be >0.5 mm for successful injection. Scale bars in A: 1 cm, B: 0.1 mm.

**CRITICAL:** The shape of the glass pipettes is critical for successful injection. A thin, straight, long taper on the pipette helps with successful injection (Figure 1). In this protocol, the tip of the pipette must pierce the skull and the skin of the neonatal pup. Thus, a quartz capillary was selected. It is necessary to use a laser puller because the melting point of quartz glasses is higher than that of borosilicate glasses. Although borosilicate glass pipettes can be used, quartz glass pipettes reduce the possibility of breakage during insertion. A conventional micropipette puller can be used if borosilicate glass pipettes are used.

4. Cut and polish the tip of glass pipettes for injection.
  a. Cut the tip of the glass pipette to obtain an outer diameter of 50 μm and an inner diameter of 20 μm using forceps and a microforge.
  b. Polish the tip of the glass pipette using a micro grinder, so it is beveled at 45°.
5. Mark the glass pipette at 2.82 cm intervals with a pen.

***Note:*** We used a 4 μL injection volume per animal in this study (step 2). A capillary length of 1.41 cm was used for a 1 μL volume because the inner diameter of the quartz capillary used in this study was 0.3 mm. To obtain a volume of 4 μL, a length of 5.68 cm was used.

### Preparation of double coverslips for cranial window

**Timing: 1–2 h**

**Figure 2:**
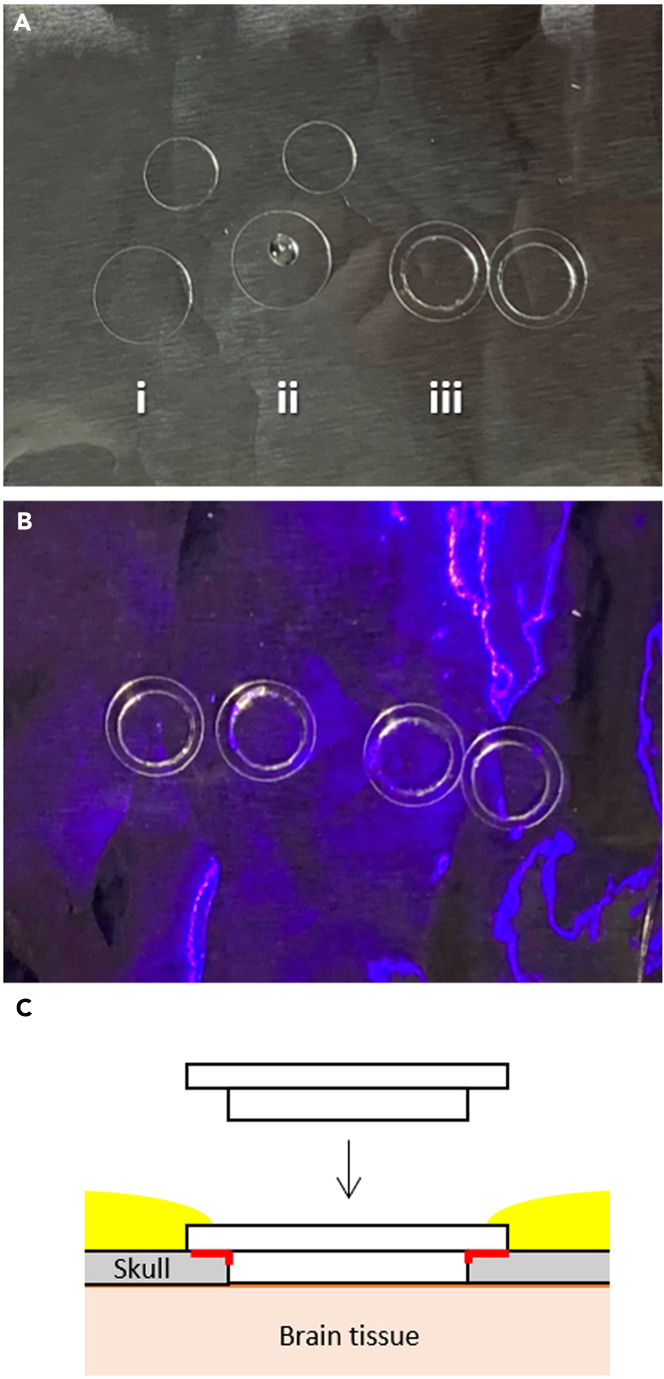
Preparation of double coverslips for the cranial window (A, i) Prepare the φ4.5 mm and φ6.0 mm coverslips on an aluminum foil. (A, ii) Apply a small drop of a UV-curing resin to the center of the larger coverslip. (A, iii) Place the smaller coverslip onto the center of the larger coverslip. (B) Apply UV light for 1 h. (C) A cross-sectional illustration showing the implantation of the double coverslip for the cranial window. Red lines indicate the cyanoacrylate adhesive applied at step 12c. Yellow areas indicate the dental cement applied at step 12d.
7. Apply a small drop of a UV-curing resin (NOA81) to the center of the larger coverslip (Figure 2A, ii).
8. Place the smaller coverslip onto the center of the larger coverslip (Figure 2A, iii) and gently push it. Ensure that there are no air bubbles.
9. Apply UV light for 1 h (Figure 2B).
10. Store the prepared double coverslips in an enclosed container at 20°C–25°C to avoid dust until use.

***Note:*** In this study, we used double-coverslip slides for the cranial window for wide-field two-photon imaging. To obtain a field-of-view (FOV) of 3 × 3 mm^2^, the imaging area of our wide-field two-photon imaging system, FASHIO-2PM, a coverslip of at least φ4.5 mm in diameter must be used. Making such a large cranial window is relatively difficult because of the curvature of the brain tissue. If a single coverslip is instead used for the cranial window, it may accidentally fall into the cranial hole when it is pushed to fully adhere to the brain tissue. The double coverslip makes it easier to form the cranial window because the edge of the larger coverslip remains on the edge of the cranial hole. This also helps fix the cranial window well with dental cement (Figure 2C). Moreover, as the experimenter does not have to worry about the cranial window falling during this procedure, the cranial window can be successfully pushed to be fully attached to the brain tissue, thus reducing tissue movement during two-photon imaging.

6. Prepare the same number of φ4.5 and φ6.0 mm coverslips on a piece of aluminum foil (Figure 2A, i).
  a. Clean the coverslips by soaking them in ethanol and drying them on a clean piece of paper.
  b. Place the cleaned coverslips on a piece of aluminum foil using forceps.

## Key resources table

**Table:** 

REAGENT or RESOURCE	SOURCE	IDENTIFIER
**Bacterial and virus strains**		

AAV-DJ-Syn-G-CaMP7.09-WPRE	(Ota et al., 2021)	N/A
AAV9-Syn-GCaMP6f-WPRE-SV40	Penn Vector Core	AV-9-PV2822

**Chemicals, peptides, and recombinant proteins**		

Fast Green FCF	Nacalai Tesque, Inc.	15939-54
Isoflurane	Pfizer	N/A

**Experimental models: Organisms/strains**		

Mouse: C57BL/6JJmsSlc	Japan SLC, Inc.	N/A

**Software and algorithms**		

ImageJ	(Schneider et al., 2012)	https://imagej.nih.gov/ij/
Falcon	Nikon Corp.	http://nikon.com/
MATLAB (versions:2018b-2021a)	The MathWorks	https://jp.mathworks.com/
NIS-elements	Nikon Corp.	http://nikon.com/

**Other**		

Laser puller	SUTTER INSTRUMENT	P-2000
Quartz capillaries without filament	SUTTER INSTRUMENT	Q100-30-15
Microforge	NARISHIGE	MF-830
Micro grinder	NARISHIGE	EG-401
Stereotaxic Micromanipulator	NARISHIGE	SM-15R
Stereo Microscope	Olympus	SZX7
Neonatal mouse head holder	NARISHIGE	Custom-made (N/A)
Small animal warmer & thermometer	Bio Research Center	BWT-100A
Flexible arm LED light	AS ONE	PF-D
Saline	Otsuka	035061311
Phosphate buffered saline (PBS) (10×)	Nacalai Tesque, Inc.	27575-31
5-mL syringe	Terumo	SS-05SZ
TygonR laboratory tubing, LMT-55 φ0.79 × φ2.38 mm	Saint-Gobain	ACFJ0001
Fluorescence macroscope	Brain Vision	THT macroscope
PLANAPO 1.6×	Leica Microsystems	10450029
PLANAPO 1.0×	Leica Microsystems	10447157
Blue LED light	Brain Vision	LEX2-B
Neutral density filter	Thorlabs	NE06B or NE13B
CMOS camera	Hamamatsu Photonics K. K	ORCA-Flash4.0 C11440-22CU
Xylocaine pump spray 8%	Aspen Japan	N/A
Surgical tape	NICHIBAN	KP158
Silk Suture with Sterile Needle	Nesco	ER2004SB45
φ22-mm micro cover glass	Matsunami	C022001
Isoflurane vaporizer	Shinano	SN-487-OT Air
Dental handpiece	Shofu	Tas-35LX
Miniature Carbides Ball Shape Burs	Foredom Electric Co.	A-212
Enrofloxacin (Baytril)	Bayer Healthcare	08713254
Dental cement, Super-Bond C&B Kit	Sun Medical	N/A
Head holder	NARISHIGE	SG-4N
φ4.5 mm coverslips (No.2)	Matsunami	Custom-made (N/A)
φ6 mm coverslips (No.1)	Matsunami	Custom-made (N/A)
Fast Curing Optical Adhesive	Thorlabs	NOA81
T-Cube LED Driver	Thorlabs	LEDD1B
385-nm Fiber-coupled LED	Thorlabs	M385FP1
Stainless-steel head plate	ExPP Co., Ltd.	Custom-made (N/A)
Bonn Micro Probe	Fine Science Tools	10030-13
Neo-Medrol EE Ointment	Pfizer	1319807M1025
Aron alpha A (cyanoacrylate adhesive)	Daiichi Sankyo Company	081019311
Dental etching liquid	SUN MEDICAL CO., LTD	204610461
Wide-field two-photon microscopy (i.e., FASHIO-2PM)	(Ota et al., 2021)	N/A
Head plate mount	ExPP Co., Ltd.	Custom-made (N/A)

## Materials and equipment

**Table:** Laser puller setting

Heat	Filament	Velocity	Delay	Pull
900–975	5	50	145	240

The ramp test value in our laboratory was 890–900. The setting parameters depend on ramp test values.

Glass pipettes can be stored in an enclosed container to avoid dust at 20°C–25°C for several months.

**Table:** Normal Rat Ringer’s solution (NRR)

Reagent	Final concentration	Amount
NaCl	135 mM	7.88 g
KCl	5.4 mM	0.40 g
MgCl_2_-6H_2_O	1 mM	0.204 g
CaCl_2_-2H_2_O	1.8 mM	0.265 g
HEPES	5 mM	2.38 g
**Total**		**1 L**

Use distilled water. Adjust pH to 7.2 with NaOH. The final osmolarity was approximately 290 mOsm/L. The solution can be stored at 4°C for 1 month. Bubble with O_2_ gas before use.

## Step-by-step method details

**CRITICAL:** All animal experiments must be performed according to the guidelines of the institution. For each step, the type of drug or anesthetic used should be modified according to the applicable guidelines.

### Neonatal AAV injection

**Timing: 15 min/animal**

**Figure 3:**
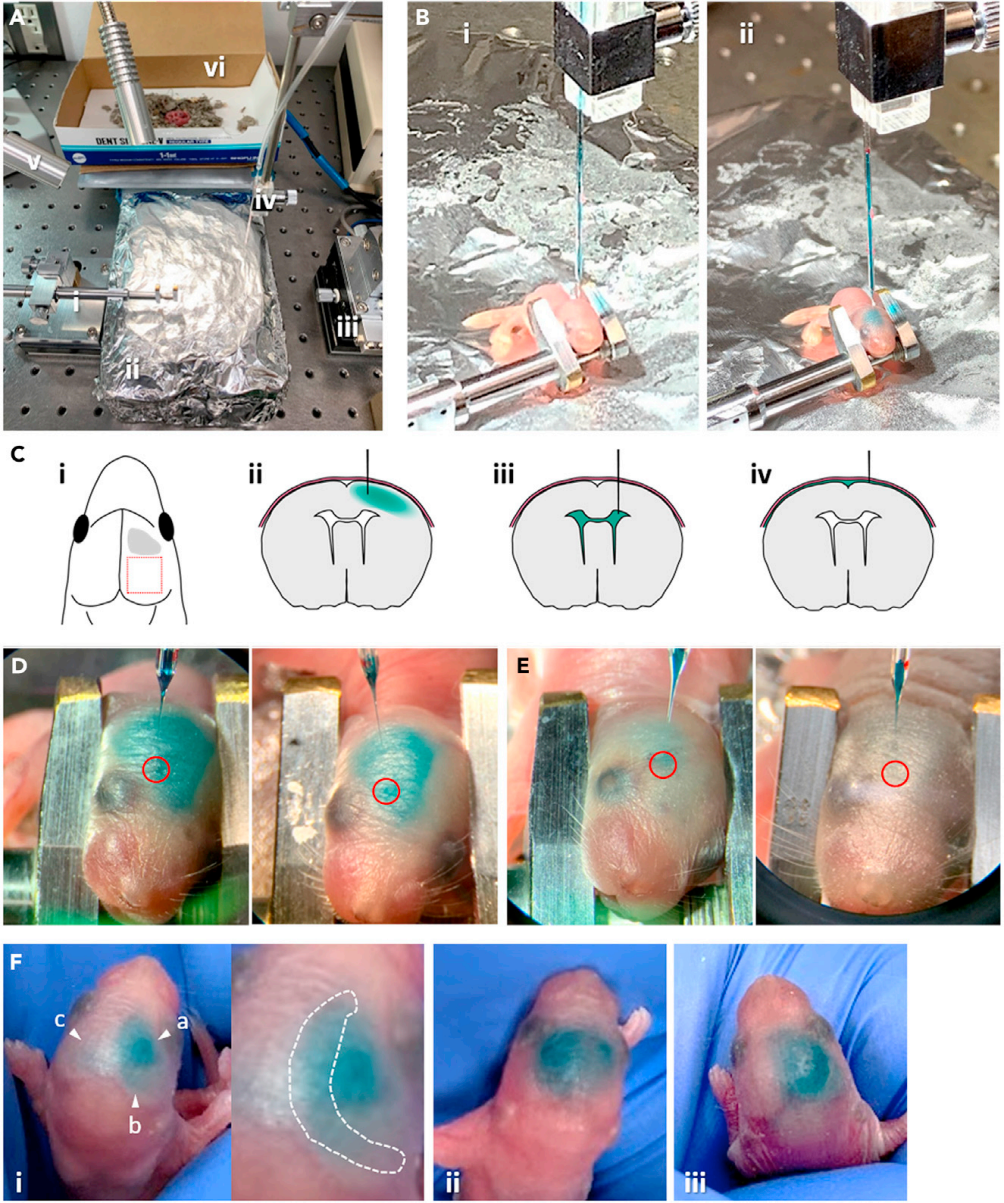
Neonatal AAV injection (A) The working space setup for neonatal AAV injection: (A, i) Head holder for pups. (A, ii) Ice bed. (A, iii) Stereotaxic micromanipulator. (A, iv) Glass pipette containing AAV solution connected with a TygonR laboratory tubing. (A, v) Light. (A, vi) Recovery box. (B) The scene before (i) and after (ii) the injection of AAV solution into a pup. (C) Injection: (C, i) The injection area in this study is shown in gray. The red square indicates a planned two-photon imaging area. (C, ii) The coronal view of the correct injection depth. The tip of the glass pipette stops in the middle of the cortical layer, so that the dye (shown in green) slowly spreads throughout the cortex. (C, iii) Coronal view of a failed injection: too deep injection depth. The pipette tip reaches the lateral ventricles (LV), so the dye quickly spreads in LV. (C, iv) Coronal view of a failed injection: too shallow injection depth. The pipette tip does not penetrate the cortical layer, so the dye accumulates between the skin and the brain tissue. (D) Examples of successful injection. The dye spreads throughout the hemisphere and does not leak to the opposite hemisphere after the injection. Red circles indicate the injection point for each pup. (E and F) Examples of failed injection. (E) The dye did not spread throughout the hemisphere. Red circles indicate the injection point for each pup. (F, i) An example of an injection point that was too deep. The dye was observed at three locations: (a) near the injection point, (b) at the right hemisphere forming a crescent shape, and (c) at the left hemisphere forming a crescent shape. The crescent formation is often observed when the pipette tip reaches the LV. Inset is the magnified view. Dashed line indicates the crescent shape, which is the result of the loaded dye accumulating in LV. (F, ii) An example of an injection point that was too shallow. The tip of the glass pipette did not reach the cortex layer and stayed beneath the skin, causing the AAV solution to accumulate between the skull and the skin, leading to skin swelling. (F, iii) An example of an injection using a pipette with a thick tip. Although the dye spreads throughout the hemisphere, the AAV solution overflows under the skin when the injection pipette is withdrawn because the tip used was too thick. See also Methods video S1.

Cortical-wide Ca^2+^ sensor (G-CaMP7.09) expression is induced in neonatal mice via quick AAV injection (Figure 3).

Our protocol was not based on a method using a microsyringe pump but on the manual injection method, which was modified from Kim et al. (2013), (2014), and Murayama and Larkum (2009).

***Note:*** Neonatal AAV injection can be performed on pups at postnatal days 0–5 (P0-5), although our recommendation is at P1-2 for reproducible results. The difficulty level of the injection increases as the pups’ age progresses because the appropriate time for anesthesia administration is dependent on the pups’ weight when using pups >P3.

**Figure 4:**
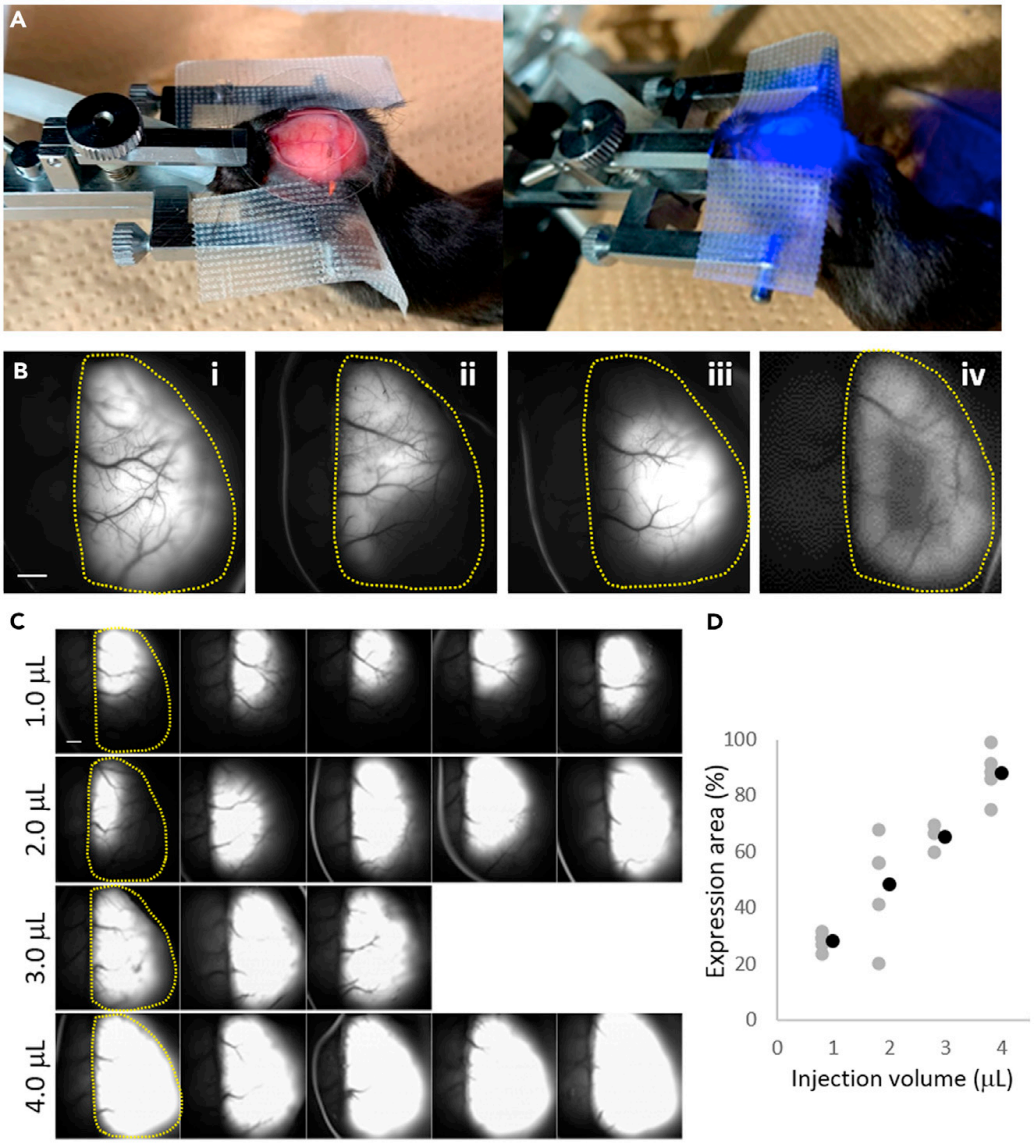
Transcranial expression check (A) The setting of transcranial expression check. Left: The mouse was set in a head holder under the macroscope for transcranial fluorescence imaging. Right: During fluorescence observation. (B) Examples of G-CaMP expression. (B, i) Successful expression: the sensor is expressed throughout the hemisphere, and the brightness of the sensor is good. (B, ii-iv) Examples of failed expression. (B, ii) The brightness of the sensor is good, but the sensor expression pattern is not good. The lateral part of the cortex lacks the sensor expression. (B, iii) The brightness of the sensor is good, but the sensor expression pattern is not good. The sensor expression is not well spread and accumulates in the parietal part of the cortex. (B, iv) The brightness of the sensor is weak, and the parietal part of the cortex lacks sensor expression. Yellow dashed lines indicate the cortex region of the injected side. (C) Fluorescence images of sensor expression after systematic testing with different injection volumes during neonatal AAV injection. Yellow dashed lines in the first column in each condition indicate the cortex region of the injected side. (D) Quantification of sensor expression in the cortex in panel (C). The expression area (y-axis) is plotted against the injection volume (x-axis) and was calculated (see also the “quantification and statistical analysis” section) by dividing the sensor expression area (the number of pixels with fluorescence intensity above the threshold) by the area of the cortex region (the number of pixels within the yellow dashed line; the cortex region was manually selected for each brain). Gray dots indicate the expression area for each brain. Black dots indicate the average of each condition. Scale bars: 1 mm.

**Figure 5:**
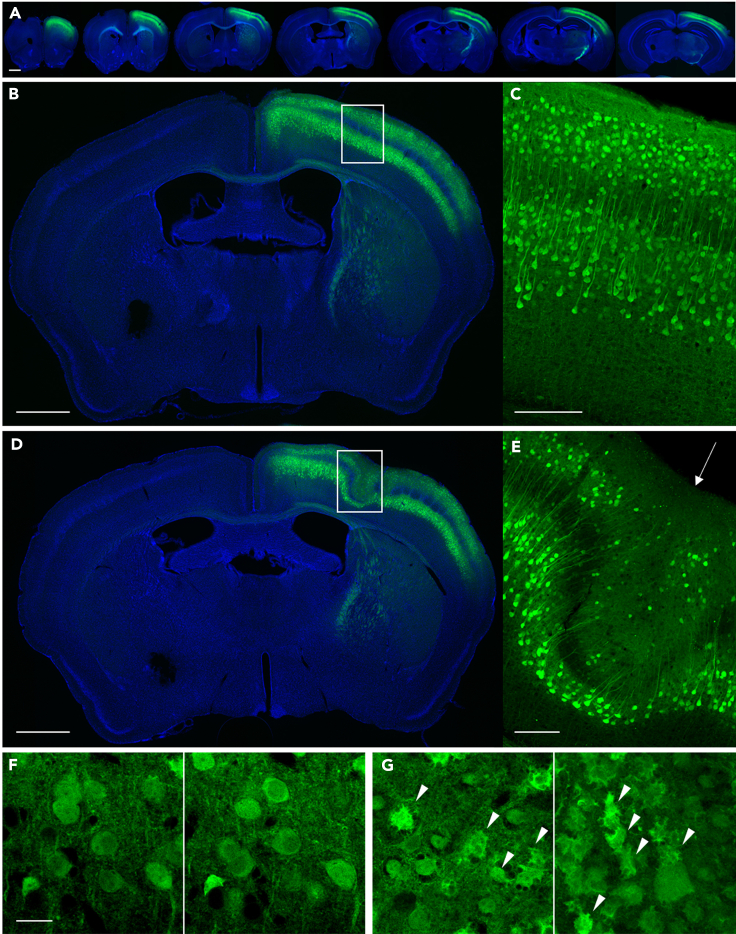
Expression check of the brain slice (A) Fluorescence images of a series of coronal sections of the brain with successful AAV injection. Sensor expression (green) can be observed from the anterior to the posterior part of the brain. Nuclei were counterstained with DAPI (blue). (B and C) Fluorescence images of a coronal section of the brain with successful AAV injection. G-CaMP7.09 signal (green) can be observed in the layer 2/3 and 5 neurons of the right hemisphere. More than 90% of the excitatory neurons in layer 2/3 were labeled with G-CaMP7.09. Nuclei were counterstained with DAPI (blue). (C) A magnified view of the boxed area in (B). (D and E) Fluorescence images of a coronal section of the brain injected with AAV using a thick glass pipette. A disrupted layer structure was observed. (E) A magnified view of the boxed area in (D). The arrow indicates the injection point. (F and G) Confocal images of brain slices. (F) The morphology of G-CaMP7.09-expressing neurons is normal. G-CaMP7.09 signal is observed only in the cytoplasm. (G) The morphology of some G-CaMP7.09-expressing neurons (marked with arrowheads) is disrupted due to the toxicity caused by high titer of AAV. Scale bars in A, B, and D: 1 mm, C and E: 200 μm, F: 20 μm.

1. Preparation of AAV, equipment, and working space (Figure 3A).
  a. Prepare AAV at a working concentration.
    i. Thaw the tube of AAV stock on ice.
    ii. Dilute AAV with saline or PBS solution. Mostly, our working titer is 1.0 × 10^11^ to 1.0 × 10^13^ GC/mL.
    iii. Add 0.1% (w/v, weight/volume) Fast Green FCF to the AAV solution to help visualize the injected area.
    iv. Maintain the AAV solution on ice until use.
  b. Prepare the working space.
    i. Prepare an ice bed, i.e., an icebox filled with ice, and cover it with aluminum foil. The pups are anesthetized by hypothermia by laying them on the ice bed.
    ii. Set the polished glass pipette to the micromanipulator and connect the pipette and a 5-mL syringe with a TygonR laboratory tubing.
    iii. Set a head holder for the pups (Figure 3A, i) and the icebox under a stereomicroscope.
  c. Bring pups to the working space.
    i. Prepare a recovery box with bedding.
    ii. Take half the number of pups from their mother and place them into the recovery box.
    iii. Place the recovery box onto the heating mat.
      **CRITICAL:** Prepare a recovery box with bedding from the home cage, which can help increase the survival rate of the pups after injection (Figure 3A, vi). Leave at least two pups with their mother during AAV injection. If all pups are taken at once, the mother will neglect them once they are returned. We usually take half of the total number of pups for the first round of injections and keep the rest of the pups with their mother. Then, we perform the next round of injections for the remaining pups. Thus, AAV injection can be performed on all littermates.

2. AAV injection in the pups. Troubleshooting 1
  a. Anesthetize a pup by laying it on top of the ice bed for 1–2 min.
    i. Anesthetize the pup until it does not move.
      ***Optional:*** When you are working on postnatal day 3–5 pups, it takes more time to anesthetize them, i.e., 3–6 min on the ice bed.
  b. Load 4.1–4.2 μL of AAV solution into the glass pipette using a syringe.
  c. Place the head of the anesthetized pup in a head holder to inject the pipette perpendicular to the cortical layer.
    i. Rotate the pup’s head slightly in the head holder, so that the injection point is facing up (Figure 3B, i).
      **CRITICAL:** It is important to set the pipette perpendicular to the cortical layer to accurately monitor the depth of the injection point and ensure safe injection. If the pipette is inserted at any other angles, the glass pipette can neither penetrate the skin nor skull and could break.
      ***Note:*** Generally, the layer structure of the injection site will not be disrupted by neonatal AAV injection (Figures 5B and 5C); however, if this occurs, we recommend avoiding the planned area for two-photon imaging. In our study, we planned to perform two-photon imaging studies in the parietal cortex, including the somatosensory area; thus, we chose the frontal region as the injection site (Figure 3C, i).
  d. Manually inject AAV into the cortex (Figures 3B–3D, Methods video S1)
    i. Set the tip of the glass pipette to the surface of the injection point.
    ii. Insert the glass pipette 280–300 μm deep from the surface.
      **CRITICAL:** The injection depth in the brain is critical for the successful spread of the dye/AAV throughout the cortex (Figures 3C–3F). If the injection depth is too deep, the tip of the pipette will reach the lateral ventricles (LV), and dye spread in a crescent formation will be observed after injection (Figures 3C, iii and 3F, i; Kim et al., 2013, 2014). If the depth is too shallow, the tip of the pipette will not reach the cortex and stay just below the skin, causing the AAV solution to accumulate between the skull and the skin, leading to skin swelling (Figures 3C, iv and 3F, ii).
    iii. Manually inject 4 μL of AAV solution into the cortex at a speed of 4 μL/min by slowly pushing the syringe. If the injection is successful, the green dye slowly spreads throughout the hemisphere (Figures 3C, ii and 3D).
      ***Note:*** The manual injection consists of two shots: the first shot is the injection of a small amount (∼300 nL), and the second shot is the injection of ∼ 3.7 μL of AAV solution (i.e., 100–200 nL solution is left in the pipette). The purpose of the first shot is to check whether the injection depth is correct. When it is correct, the dye is observed as a small dot at the injection site after the administration of the first shot (within 10 seconds after the start of injection, 00:09–00:10 in Methods video S1). When a small dot is not observed, slight adjustments should be made to find a better depth at which a small dot of the dye appears following the injection of a small amount of the AAV solution. After confirmation, a second shot with higher volume (3–4 μL) is performed. When the injection is successful, the dye will slowly spread throughout the hemisphere and will not leak into the opposite hemisphere (00:20–00:24 in Methods video S1, Figure 3D).
      ***Note:*** The area and brightness of sensor expression across the cortex depend on the volume of AAV solution injected (Figure 4C). We found that the injection of 4 μL AAV solution per animal achieved consistent results for cortical-wide expression. We did not find any developmental differences between the pups injected with 4 μL of AAV solution and those without. In addition, the injection of the 4 μL AAV solution was effective even at different injection depths. In step 2d-iii, if the initial spread of the injected dye appears to be different, the experimenter can adjust the depth of the injection to find the correct depth.
    iv. Withdraw the glass pipette from the brain.
  e. Recover the pups.
    i. Remove the pup from the head holder and place it inside the recovery box on top of a heating mat.
      **CRITICAL:** To avoid low recovery rates, the injection procedure (steps 2a-e) must be performed no longer than 10 min per pup.
  f. Repeat steps 2a-e for each pup in the recovery box.
    i. Reuse the pipette until it breaks or is clogged.
  g. Gently return the injected pups to their mother with the bedding.
    **CRITICAL:** Check the temperature of the pup by touching it before returning to its mother. Returning a pup with a low body temperature results in the mother neglecting it, which reduces survival rate. Usually, warming the pups for 10 min inside the heated recovery box is sufficient to achieve the normal body temperature.
    ***Note:*** In this protocol, we introduced an AAV solution to the brain by manual injection. This results in the quick completion of the AAV injection for each pup. Quick AAV injection increases the rate of recovery from hypothermia and provides high-throughput results when comparing several sensor conditions.
    ***Note:*** We found that the AAV-DJ serotype is useful for wide and dense sensor expression in the cortex. The other option is the AAV8 serotype, which can also be used for wide and dense sensor expression. AAV9 is the third option; however, its expression is sparse compared with that of AAV-DJ or AAV8.
    ***Optional:*** You can mix at least two different AAVs for different sensor expressions in one injection. In addition, the combination of transgenic mouse lines and Cre-dependent viral vectors can be used to label specific cell types.
    ***Optional:*** This neonatal AAV injection method can be applied not only for calcium indicators but also for any other proteins or indicators of interest, such as voltage indicators or optogenetic tools.

Methods video S1. Neonatal AAV injections The video is played at 4× speed, related to step 2

### Maturation of pups and AAV maturation

**Timing: 3–4 weeks (dependent on experimental design)**

The injected pups are kept in their home cage until a Ca^2+^ sensor is expressed in the cortex well.

3. Wait for a predetermined time to allow the animals to grow and for sufficient G-CaMP7.09 (or your Ca^2+^ sensor) expression for imaging. Troubleshooting 2 and 3

***Note:*** Successful AAV induction in cortical neurons did not affect pup maturation, behavior, or survival. We found that more than 90% of the injected pups matured without any problems.

***Optional:*** If you use a tamoxifen-induced transgenic line (e.g., Cux2ERT), apply tamoxifen when pups are more than 4 weeks old. Wait for another 2 weeks to allow tamoxifen-induced gene expression.

### Transcranial expression check

**Timing: 30 min/animal**

The Ca^2+^ sensor expression is checked using transcranial fluorescent observation. Usually, we wait for 4 weeks to allow the infected cells to express G-CaMP after AAV injection (Figure 4).

***Note:*** This step confirms whether the neonatal AAV injection worked well by observing the spread of the sensor expression in the cortex and its fluorescence intensity, which correlates with the expression level.

4. Anesthetize mice with 0.5–2% isoflurane.
5. Set the mouse to the head holder and expose the skull.
  a. Set the head of the mouse in a head holder.
  b. Shave hair and clean the skin with 70% EtOH.
  c. Spray local anesthetic to the skin.
  d. Cut the scalp and expose the skull.
  e. Attach the cut scalp to the left and right sides with surgical tape.

6. Observe the sensor fluorescence with fluorescent microscopy. Troubleshooting 4
  a. After exposing the skull, apply saline to prevent drying and place a φ22 mm coverslip on the observation area.
    ***Note:*** The use of a coverslip keeps the saline solution from drying out or falling off from the skull surface during imaging.
  b. Set the mouse under fluorescent microscopy and observe the green fluorescence with blue light. If AAV injection is successful, green fluorescence can be observed throughout the hemisphere.
    ***Note:*** The wavelength of the excitation light depends on the Ca^2+^ sensor used. In our study, we used a 465 nm (center wavelength) LED.
    ***Note:*** The macro-imaging parameters that will be used to assess the expression level depend on the experimental devices. We recommend systematically recording the fluorescence intensities under several conditions, such as different emission light powers, detector gains, and exposure times during macro-imaging until the experimental conditions are fixed. The optimal macro-imaging conditions should be determined retrospectively from the results of two-photon imaging. We used macro-imaging conditions from an animal that showed dense sensor expression (i.e., cortical-wide expression and high fluorescent intensity) and Ca^2+^ signals with good signal-to-noise ratios (SNR) in the whole FOV during two-photon imaging.
    **CRITICAL:** When observing the expression, we confirmed that green fluorescence spread throughout the hemisphere with the appropriate brightness (Figure 4B). If the brightness is dark, the expression is insufficient.

7. Stitch up the skin and recover the mouse.

***Optional:*** Sensor expression can also be observed in a brain slice preparation (Figure 5). Successful injection results in a dense and uniform distribution of G-CaMP7.09-expressing neurons in the cortex. We found that 85.1–90.2% of all layer 2/3 (L2/3) excitatory neurons in the injected cortical areas could be labeled through successful injection (Ota et al., 2021). If disrupted layers are observed at the injection point, the diameter of the glass pipette used may be too thick. Damaged cells around the injection area indicate that the AAV titer used is too high (Figure 5G).

### Maturation of mice

**Timing: 3–4 weeks (depends on experimental design)**

8. Wait for a predetermined time to allow the animals to grow for craniotomy.

***Note:*** We usually wait until the mice are more than 6 weeks old.

### Craniotomy and head plate implantation

**Timing: 3–5 h/animal**

The double coverslips and a head plate are implanted to allow two-photon imaging. The surgical operation of the implantation basically follows previously reported protocols. (Please see the following references for more in-depth methodological details: Goldey et al., 2014; Holtmaat et al., 2009; Maruoka et al., 2017).

***Note:*** The location of the cranial window depends on the experimental design (i.e., imaging region, such as frontal or parietal areas). In our study, we planned to perform two-photon imaging in the parietal cortex including the somatosensory area, so we set the center position of the cranial window to 0.7–0.8 mm posterior and 1.5–2.0 mm lateral of the bregma.

**Figure 6:**
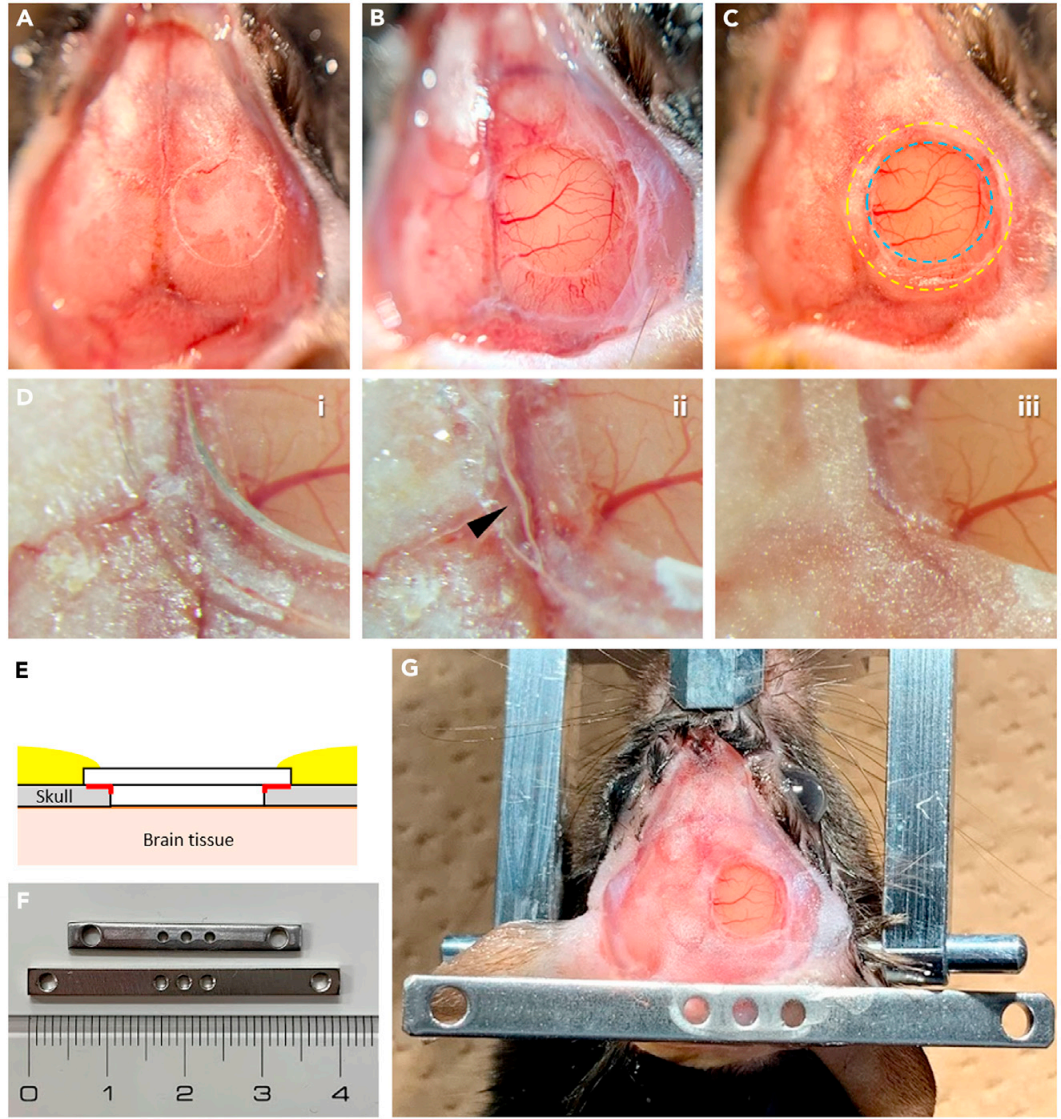
Craniotomy and head plate implantation (A) Expose the skull and scratch the surface of the skull along the cranial hole using a fine needle. (B) Gently drill a cranial hole along the mark and remove the dura mater. The surface of the brain tissue is covered with NRR. (C) Place and fix the double coverslip into the cranial hole. The blue dashed circle indicates the smaller coverslip; the yellow dashed circle indicates the larger coverslip. (D) Magnified views of the placement of the double coverslip. (D, i) Place the double coverslip into the cranial hole. (D, ii) Apply a thin layer of cyanoacrylate adhesive (Aron Alpha) between the edge of the larger coverslip and the skull edge. The arrowhead indicates the edge of the applied cyanoacrylate adhesive. (D, iii) Fix the coverslips to the skull using a dental cement solution. (E) A cross-sectional illustration showing the implantation of the double coverslip for the cranial window. Red lines indicate the cyanoacrylate adhesive applied at step 12c. Yellow areas indicate the dental cement applied at step 12d. (F) Stainless bars used in this study. The thickness of the stainless bar is 1.5 mm. (G) Place and fix a stainless head plate horizontal to the surface of the coverslips using dental cement. The cement entirely covers the exposed skull, wound margins, and edges of the dental cement used to fix the coverslips and the head plate.
  b. Fix the plate onto the skull with the dental cement and allow it to cure.
  c. Apply dental cement to entirely cover the exposed skull, wound margins, and the edges of the dental cement used to fix the coverslips and the head plate and allow it to fully cure (Figure 6G).

***Note:*** We recommend waiting for 20–30 min to allow the dental cement to fully cure, so that the head plate is firmly fixed.

9. Anesthetize the mouse with an anesthetic of choice (e.g., isoflurane).
10. Administer antibiotic enrofloxacin (5 μg/g body weight) via subcutaneous injection.

11. Create a cranial window large enough for the FOV of the two-photon microscopy.
  a. Set the head of the mouse in the head holder.
  b. Shave hair from the surgical site and clean the skin with 70% EtOH.
  c. Cover the eyes of the mouse with cream to prevent dryness.
  d. Apply a topical anesthetic to the skin.
  e. Cut the skin and expose the skull.
  f. Clean the exposed skull with a dental etching liquid.
  g. Gently scratch the surface of the skull along the cranial hole using a Bonn Micro Probe, a fine needle (Figure 6A).
    ***Note:*** Because the FOV of FASHIO-2PM is 3 × 3 mm^2^, we used a φ4.5 mm coverslip that covered the entire FOV in this study. The cranial hole should be slightly larger than the diameter of the smaller coverslip to accommodate the double coverslip implant.
  h. Drill a groove along the mark made in step 11-g using a drill.
    ***Note:*** To avoid heat damage to the brain tissue, we recommend using a drill speed of 1000–5000 rpm. It takes roughly 30–60 min to complete drilling along the large mark (diameter 4.5 mm) of the cranial window. Remove small bone fragments or dust generated during drilling using air duster or suction. We recommend opening the cranial window very gently. The groove for a craniotomy of φ4.5 mm may overlap with the cranium joint, and this can easily cause bleeding if the groove is drilled roughly. Thus, gentle drilling is recommended to prevent bleeding during a large craniotomy.
  i. Apply NRR to the groove, gently remove the island of the grooved skull by lifting it with fine forceps, and expose the dura mater.
    ***Note:*** It takes approximately 10–20 min to completely remove the skull with no bleeding. Apply NRR constantly so that when removing the skull, the dura mater can be easily detached. If the skull is removed before the dura mater is detached, bleeding from the dura will occur. If bleeding occurs, rinse the surface of the dura with the NRR solution until it stops.
  j. Rinse the surface of the brain tissue with NRR.
    **CRITICAL:** The surface of the brain tissue should be kept moist by applying NRR constantly until the completion of the double coverslip implantation. Drying of the surface causes damage to the tissue.
  k. Remove the exposed dura mater with fine forceps (Figure 6B).
    ***Note:*** It is better to leave the dura mater intact because its removal may cause microglial activation in some cases. In our study, transient activation of microglia was observed by removing the dura. This activation peaked at 2 weeks post-surgery and returned to the normal level after up to 6 weeks post-surgery (Ota et al., 2021; Figures S13A–S13C). We usually wait for more than six weeks to conduct chronic two-photon imaging experiments (Ota et al., 2021).
    In general, the presence of the dura mater interferes with the efficiency of the two-photon excitation and the collection of emission light from the specimens, which reduces the SNR. Moreover, the excitation time per pixel (called dwell time) is shorter in wide-field and high-speed imaging systems than in conventional two-photon systems. This shortening of dwell time also decreases the SNR. Therefore, the dura mater was removed in our study to improve the SNR.

12. Implant the double coverslip on the skull surface (Figures 6C–6E).
  a. Rinse the surface of the brain tissue and the double coverslip with NRR.
  b. Place the double coverslip onto the cranial hole (place the smaller coverslip into the cranial hole, so that it is in contact with the brain surface, Figure 6E) and push it slightly to ensure that the brain adheres to the entire area of the small coverslip side (Figure 6D, i).
    ***Note:*** The edge of the larger coverslip catches on the edge of the skull, preventing the double coverslip from accidentally falling through the cranial hole while pushing it to allow it to entirely adhere to the brain tissue (Figure 6E).
  c. Apply a thin layer of cyanoacrylate adhesive (Aron Alpha) between the edge of the coverslip and the edge of the cranial hole. Allow this to dry for ∼5 min (Figure 6D, ii).
    ***Note:*** The liquid (e.g., brain fluid or NRR) prevents the dental cement from curing. The cyanoacrylate adhesive works with the liquid and allows the surface to dry. This helps to cure the dental cement properly. In addition, the cyanoacrylate adhesive prevents the dental cement solution from leaking into the gap between the coverslip and the cranial hole (Figure 6E).
  d. Seal the double coverslip to the skull using dental cement, covering the edges of the larger coverslip, and allow it to cure (Figure 6D, iii).
    ***Note:*** To minimize tissue movement during two-photon imaging, the double coverslip must be fixed so that the surface of the coverslip fully adheres to the brain surface. Therefore, after applying it to the edges of the coverslip and skull, we recommend waiting for the dental cement to fully cure, which approximately takes 10–20 min (depending on the solution used).

13. Implant a stainless head plate to the skull.
  a. Place a stainless head plate (Figure 6F) on the skull and adjust the angle horizontally to the surface of the coverslips.

14. Recover the mouse and wait for at least 6 weeks to conduct two-photon imaging experiments.

## Expected outcomes

**Figure 7:**
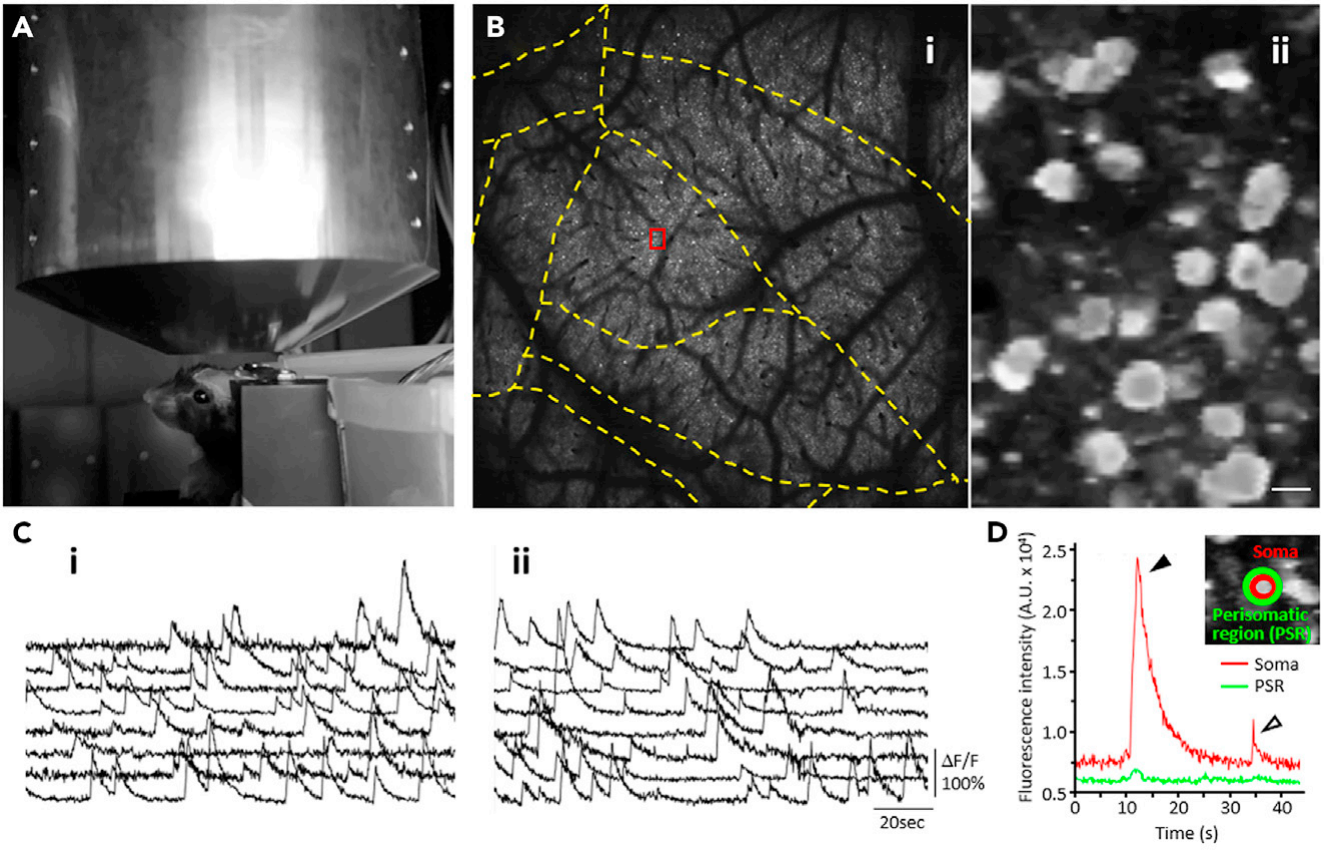
Two-photon imaging using FASHIO-2PM (A) A mouse is held under the objective lens of FASHIO-2PM using a head plate mount. (B, i) Ca^2+^ imaging from the cortical layer 2 neurons labeled with G-CaMP7.09 in a contiguous full FOV (3 × 3 mm^2^), including multiple brain regions, shown as yellow dashed lines according to the Allen Mouse Common Coordinate Framework. (B, ii) A magnified view of the area outlined by a red box in (i). (C) Representative Ca^2+^ signals from randomly selected eight neurons at acute imaging (i, 12 h after surgery) and at chronic imaging (ii, 6 weeks after surgery) are shown. (D) Representative Ca^2+^ signals at the soma (red) and fluorescence changes in the perisomatic region (PSR, green) are shown. The inset shows the region of interests (ROIs) from the soma and PSR. We usually monitor the Ca^2+^ signals with both fast (open arrowhead) and long (solid arrowhead) decay times in the soma compared with those in the PSR. Scale bar in B, ii: 15 μm.

Successful completion of this protocol allows the observation of activity from more than 10,000 neurons in layer 2 in the 3 × 3 mm^2^ FOV with FASHIO-2PM (Figure 7). A representative result and a video of Ca^2+^ signals are provided in our previous article (Ota et al., 2021). Troubleshooting 4 and 5

## Quantification and statistical analysis

The sensor expression area in the cortex in the “transcranial expression check” (Figure 4) was quantified using ImageJ software (Schneider et al., 2012). The normalized expression area was calculated as follows: E_A_/C, where E_A_ is the number of pixels of the expression area and C is the number of pixels of the cortex region in each experimental condition. The expression area was selected with fluorescence intensity above a certain threshold (set as the same value for all the conditions) using the “Threshold” tool. The cortex region was selected manually using the “Freehand selection” tool.

## Limitations

This protocol describes a quick AAV injection procedure in neonatal mice to express the gene of interest with high density throughout the cortex. It offers an alternative method to the use of transgenic animals. It also saves time because many experimental conditions, such as the types of AAVs and sensors, can be tested in a high-throughput manner because of the quick injections. However, because gene expression approximately takes 2 weeks, our protocol is not suitable to study neural activity during the developmental stage.

Although extra work is required compared with methods using transgenic animals, our protocol provides various options for functional and anatomical imaging studies, such as Ca^2+^ imaging focusing on specific cell types by combining several transgenic mouse lines and Cre-dependent viral vectors, voltage imaging using AAVs to express voltage indicators, imaging with optogenetic manipulation by combining AAVs for Ca^2+^ sensors and optogenetic tools, or imaging with anatomical tracing proteins or dyes.

## Troubleshooting

### Problem 1

Fast green dyes with AAV solution did not spread throughout the hemisphere. The AAV solution overflowed when the injection pipette was withdrawn from the brain (Step 2).

### Potential solution

The injection depth was not appropriate. When the injection depth is too deep, you can observe dye spread in a crescent formation (Figure 3F, i). Contrarily, when the depth is too shallow, the dye spreads around the injection point only, and the solution accumulates between the skull and the skin (Figure 3F, ii). Check the appearance of the dye after the first shot (injection of ∼300 nL) and adjust the depth slightly to find a better depth where a small dot of the dye will appear when a small amount of the AAV solution is injected.

If the injected solution overflows from the skin when the glass pipette is withdrawn, this could be due to one of these reasons: the tip of the pipette was thick, the tip has been broken during repeated injections for the littermates, or the pup’s head has moved during the injection. In the first and second cases, use a new glass pipette. In the third case, increase the level of anesthesia. Prolong the time for anesthesia administration in the next pup. If these two options do not work, wait for 1–2 min before withdrawing the glass pipette.

### Problem 2

Pups’ survival rate is low (Step 3).

### Potential solution

The pups might not have fully recovered after the injection before returning them to their mother. In general, mother mice stop caring for pups when they smell different or when their body temperature is low. Put some bedding from the home cage into the recovery box to retain the mother’s smell. Ensure that the pups’ body temperature has returned to normal by confirming their temperature with your hand before returning the pups to the mother’s cage.

C57BL/6 mother mice sometimes do not care for their babies. In this case, you can either take the pups to a foster mouse such as the Institute of Cancer Research (ICR) mouse or provide a covered shelter in the home cage to make the mother mouse feel safe and ensure that she takes good care of the pups.

If the survival rate does not improve after taking the abovementioned solutions, the AAV titer used might be too high for the mice; try a lower titer.

### Problem 3

Strange features in AAV-injected mice, such as hyperactivity, bristled fur, or bad growth, are observed on monitoring (Step 3).

### Potential solution

The AAV titer or the sensor that you chose may be toxic to mice. We systematically tested several combinations of promoters, sensors, and titers before determining the final conditions. We found that AAV with the synapsin promoter, G-CaMP7.09, and a working titer of 1.0 × 10^11^ to 1.0 × 10^13^ GC/mL resulted in a dense expression for two-photon imaging and did not cause toxicity in mice. Because an ideal condition will depend on the sensors and promoters you want to use, we recommend testing several conditions.

### Problem 4

The expression of G-CaMP results in sparse labeling of cortical neurons and/or in local labeling (Step 6 or Expected outcomes).

### Potential solution

First, increase the titer of the AAV used. If this does not work, change the serotype because it may not be appropriate to yield a dense and wide expression. We found that AAV-DJ, AAV8, or AAV9 works well.

### Problem 5

The cranial window becomes fogged before performing two-photon imaging, which reduces the SNR in the labeled neurons (Expected outcomes).

### Potential solution

Rinse the surface of the brain tissue well with NRR before placing the double coverslip. If pieces of shaved bone are left in the cranial hole, they will damage the brain tissue and cause bleeding or inflammation, resulting in the fogging of the cranial window.

## Resource availability

### Lead contact

Further information and requests for resources and reagents should be directed to and will be fulfilled by the lead contact, Masanori Murayama (masanori.murayama@riken.jp).

### Materials availability

All plasmids and AAVs generated in this study are available from the lead contact with a completed material transfer agreement.

## Data Availability

Data and custom-written software used for data acquisition analysis can be requested from the lead contact.
